# HER2/PD1 bispecific antibody in IgG4 subclass with superior anti‐tumour activities

**DOI:** 10.1002/ctm2.791

**Published:** 2022-04-05

**Authors:** Wendi Chu, Hui Xu, Yanfei Wang, Yongle Xie, Yi‐Li Chen, Xiaorong Tan, Chenghao Huang, Guifeng Wang, Qi Wang, Wenxin Luo, Ningshao Xia, Meiyu Geng, Zuoquan Xie, Chunhe Wang

**Affiliations:** ^1^ Biotherapeutics Discovery Research Center Shanghai Institute of Materia Medica, Chinese Academy of Sciences Bldg#1, 720 Cai Lun Road Shanghai 200126 PR China; ^2^ State Key Laboratory of Drug Research Shanghai Institute of Materia Medica, Chinese Academy of Sciences 555 Zu Chong Zhi Road Shanghai 200126 PR China; ^3^ University of Chinese Academy of Sciences Beijing PR China; ^4^ State Key Laboratory of Molecular Vaccinology and Molecular Diagnostics School of Public Health and School of Life Science Xiamen University Xiamen PR China; ^5^ Dartsbio Pharmaceuticals Ltd Zhongshan PR China; ^6^ Shanghai Mabstone Biotechnology Ltd Shanghai PR China

Dear Editor,

Monoclonal antibodies (mAbs) targeting either human epidermal growth factor receptor‐2 (HER2) or programmed death‐1 (PD1) have been successful in the clinic. However, patients develop resistance to either HER2‐ or PD1‐targeting monotherapy,^1^, ^2^ and the advantages of HER2‐targeting in breast and gastric cancers are not recapitulated in other HER2‐positive cancer types.^3^, ^4^ Notably, up‐regulation of PD‐L1 could render HER2‐positive tumours resistant to HER2‐targeted therapies, while it could be overcome by bispecific antibody (BsAb) strategies targeting both HER2 and PD‐1 pathways.^5^, ^6^ However, the HER2/PD‐1 BsAb molecules reported previously all adopted unmodified IgG1 subclass from trastuzumab. IgG1 is a potent trigger of antibody‐dependent cell‐mediated cytotoxicity (ADCC)^7^ and complement‐dependent cytotoxicity (CDC) to directly attack HER2‐positive tumour cells, but when in HER2/PD1 BsAbs, may cause collateral damage to activated PD1‐expressing T cells and thus may temper their clinical efficacy. Up to today, no HER2/PD‐1 BsAbs in IgG4 or IgG2 form, which have weakened ADCC effects compared to IgG1, have been reported.

We designed a tetravalent HER2/PD1 BsAb in IgG4 subclass in tandem IgG‐scFv (single‐chain fragment variable) format. The symmetric structure could circumvent the reported mispairing issue between heavy and light chains.^8^ Variable heavy (V_H_) and variable light (V_L_) domain sequences of anti‐HER2 Fab were adopted from trastuzumab and connected by a (GGGGS)4 linker. Anti‐PD1 antibody 5# was discovered by mouse immunization with human PD1‐expressing HEK293 cells followed by hybridoma technology, and then humanized by ‘framework shuffling’ using bacteriophage display technology (Table S1). The human IgG4 Fc containing a S288P mutation (to reduce Fab‐arm exchange) was adopted to reduce ADCC. The scFv was connected to the C‐terminus of Fc by a GGGGSGGGGTGGGGS linker.

Since the inclusion of scFv may affect the affinity and stability of BsAb molecules,^9^ we first determined which antibody, i.e., anti‐HER2 or anti‐PD1, was more suitable to be presented as scFv by comparing PD1/HER2 and HER2/PD1 BsAb molecules (Figure 1A,B). Both molecules showed comparable purities (Figure S1A,B). PD1/HER2 bound to PD1 protein with affinity comparable to pembrolizumab, while HER2/PD1 exhibited moderately reduced affinity (Figure S1C). As for HER2 binding, PD1/HER2 almost lost it completely, while HER2/PD1 was comparable to trastuzumab (Figure S1D). In addition, HER2/PD1 was more potent than PD1/HER2 in inhibiting AKT (also known as Protein Kinase B) phosphorylation (Figure S1E). Thus, compared to anti‐HER2, anti‐PD1 is more suitable to be presented as scFv in BsAb.

**FIGURE 1:**
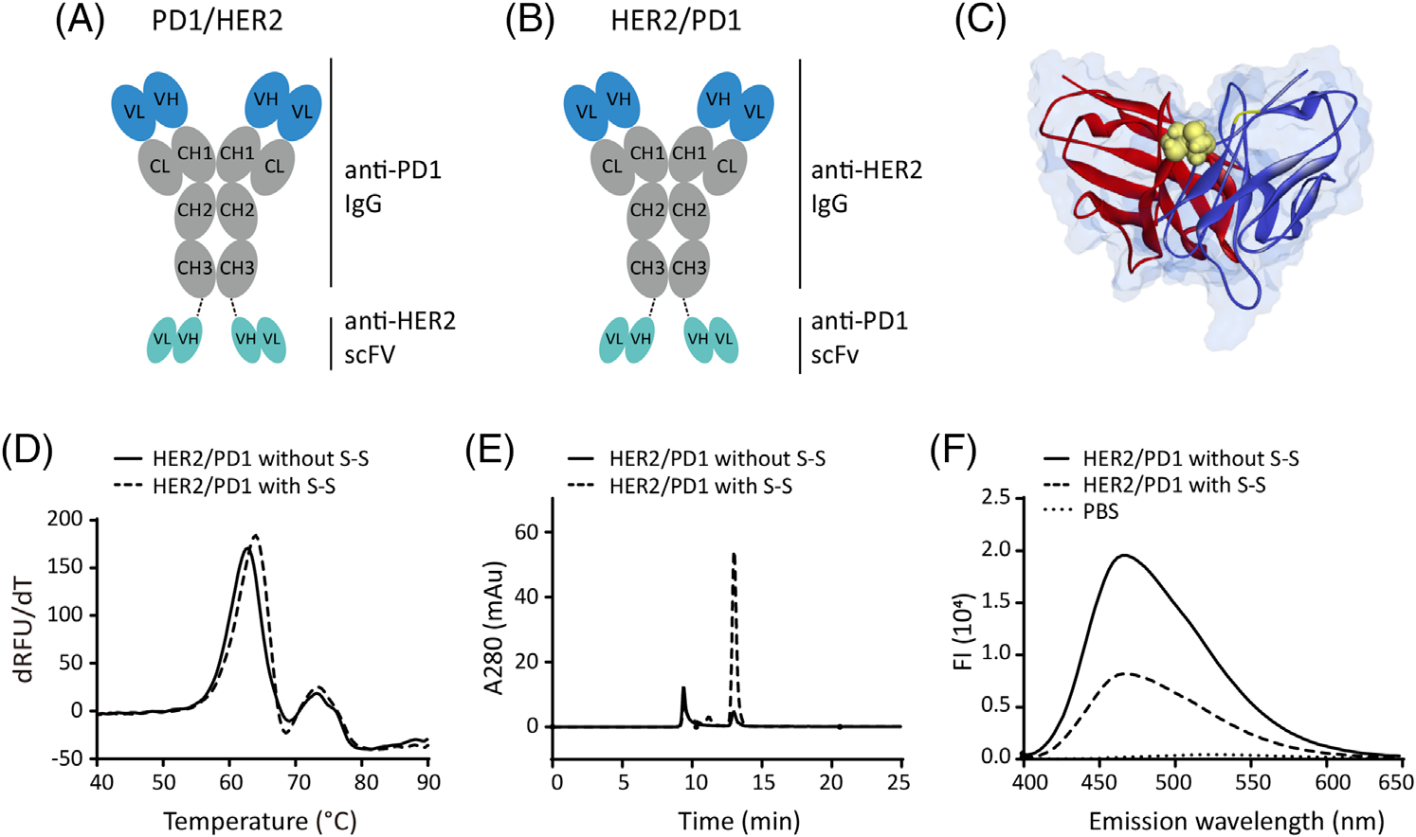
Optimization of HER2/PD1 BsAb. Structure model of (A) PD1/HER2 and (B) HER2/PD1 BsAbs. (C) Structure of scFv with V_H_44‐V_L_100 disulfide bond (S‐S, yellow balls) by homology modeling. H and L chains of scFv were shown in red and blue, respectively. (D) Differential scanning fluorimetry (DSF) analysis of HER2/PD1 BsAb with or without V_H_44‐V_L_100 disulfide bond. T_m_ values of 63.9°C (with S‐S) and 62.7°C (without S‐S) were analysed by the melting curves. (E) The purity of HER2/PD1 BsAb with or without disulfide bond was checked by SEC‐HPLC (Size Exclusion Chromatography‐High Performance Liquid Chromatography) after being heated for 1 h at 60°C. (F) The fluorescence intensity of ANS (8‐Anilino‐1‐naphthalenesulfonic acid) increased when the dye bound to the hydrophobic regions of a protein, and the degree of aggregation was inferred by the fluorescence intensity of the antibodies in pressure

To improve the stability of BsAbs, an interchain disulfide bond between V_H_44 and V_L_100 in scFv was introduced (Figure 1C). BsAbs with and without the disulfide bond were compared for thermal stability, and their *T_m_* values were determined to be 63.9°C and 62.7°C, respectively (Figure 1D). After being heated for 1 h at 60°C, BsAb with the disulfide bond showed less aggregation than the one without (Figure 1E,F). Therefore, the introduction of the interchain disulfide bond increased thermal stability.

The binding affinities (*K_D_* values) of HER2/PD1 IgG4 BsAb to HER2 and PD1 proteins were determined to be 1.0 and 2.0 nM, respectively (Figure 2A,B). BsAb also bound to HER2 and PD1 expressed on cell surface with affinities comparable to trastuzumab and anti‐PD1 5# mAb (Figure 2C,D). Additionally, it could simultaneously bind to both HER2 and PD1 (Figure 2E,F). BsAb retained the ability of anti‐PD1 5# mAb in blocking PD1 and PD‐L1 interaction, though was slightly inferior to pembrolizumab (Figure 2G). Interestingly, BsAb in IgG4 could couple PD1‐expressing CHO cells (CHO‐PD1) to HER2‐expressing NCI‐N87 cells, significantly increasing the percentage of double‐staining cell population (Figure 2H,I). Presumably, it would recruit PD1‐expressing activated T cells to HER2‐expressing tumour tissues in vivo. Treatment with BsAb or pembrolizumab resulted in concentration‐dependent increases of IFN‐γ secretion (Figure S2A). BsAb, similarly to trastuzumab, also dose‐dependently inhibited the AKT phosphorylation and proliferation of HER2‐expressing cancer cells (Figure S2B–E). Therefore, BsAb exhibited dual biological activities of both HER2 and PD1 antibodies in vitro.

**FIGURE 2:**
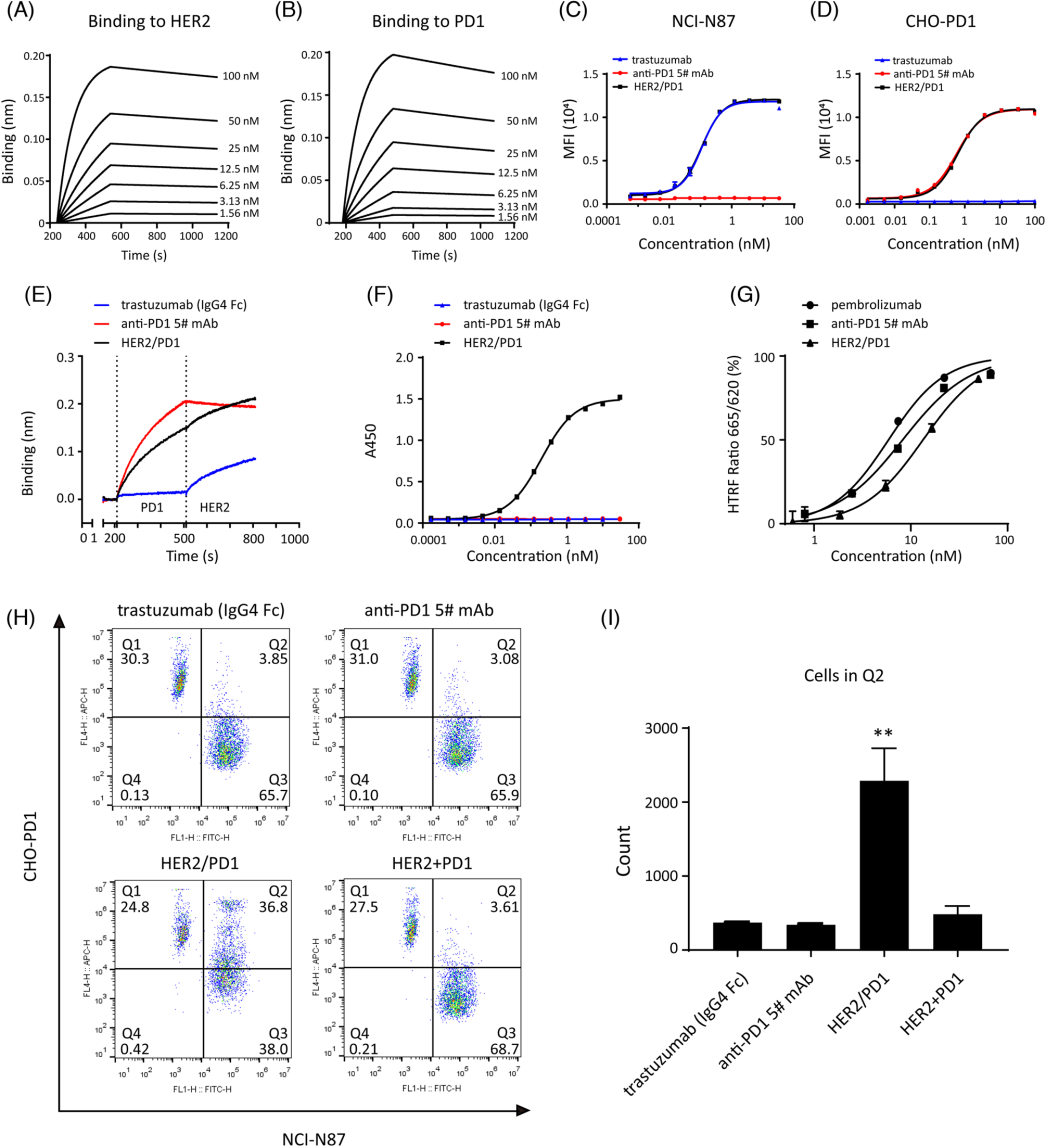
HER2/PD1 BsAb bound to HER2 and PD1 with strong affinities. The binding affinities of BsAb to (A) HER2 and (B) PD1 as detected by biolayer interferometry (BLI) assay. BsAb dose dependently bound to (C) HER2 or (D) PD1 expressed on the surface of NCI‐N87 or CHO‐PD1 cells analyzed by flow cytometry. The abilities of BsAb simultaneously bound to HER2 and PD1 proteins were determined by (E) BLI and (F) ELISA (Enzyme Linked Immunosorbent Assay) in triplicate. (G) BsAb blocked the PD1/PD‐L1 interaction in a dose‐dependent manner determined by homogeneous time‐resolved fluorescence (HTRF) assay. The *IC_50_* values calculated from dose‐response data were: HER2/PD1 (*IC_50_*: 11.3 ± 3.0 nM), anti‐PD1 5# (6.8 ± 2.0 nM) and pembrolizumab (*IC_50_*: 4.2 ± 2.4 nM). (H) HER2/PD1 BsAb could couple HER2‐expressing NCI‐N87 and PD1‐ expressing CHO‐PD1 cells. Percentages of double‐positive cell population (Q2) were examined by flow cytometry. (I) Fluorescence intensity values of all cells were collected, then double‐positive (CFSE^+^/670^+^, Q2) cells were selected and shown in the histogram. The experiment was repeated three times. Results were shown as mean ± SEM. **p < .01, by one‐way ANOVA

As expected, both BsAb and trastuzumab could suppress HER2‐positive NCI‐N87 tumour growth with comparable activity (Figure S3A), without causing obvious weight loss (Figure S3B). In an MC38 tumour syngeneic model established in human PD1 ‘knock‐in’ mice that are immunocompetent, BsAb, pembrolizumab and anti‐PD1 5# suppressed 56.9%, 40.0% and 19.1% of tumour growth, respectively (Figure S3C,D). Therefore, BsAb retained anti‐tumour activities of anti‐HER2 and surpassed anti‐PD1 in two different tumour models. Furthermore, we used HER2‐positive and trastuzumab‐resistant HCC1954 breast cancer cells, which contain a PI3KCA mutation.^10^ The expression levels of HER2 and PD‐L1 were confirmed (Figure 3A). In vitro, neither trastuzumab nor BsAb reduced the AKT phosphorylation or proliferation of HCC1954 cells (Figure 3B,C), indicating that this cell line is resistant to inhibition of HER2 signaling alone. Surprisingly, BsAb completely abolished HCC1954 tumour growth in NCG mice reconstructed with human peripheral blood mononuclear cells (PBMCs) (Figure 3D,E), while mAbs and combination treatments alone only slightly suppressed tumour growth and failed to reach statistical significance. Our result indicated that BsAbs could offer additional benefits than merely blocking HER2 or PD1 pathways in vivo. In addition, the pharmacokinetics profile of BsAb was comparable to mAbs (Figure S4A,B).

**FIGURE 3:**
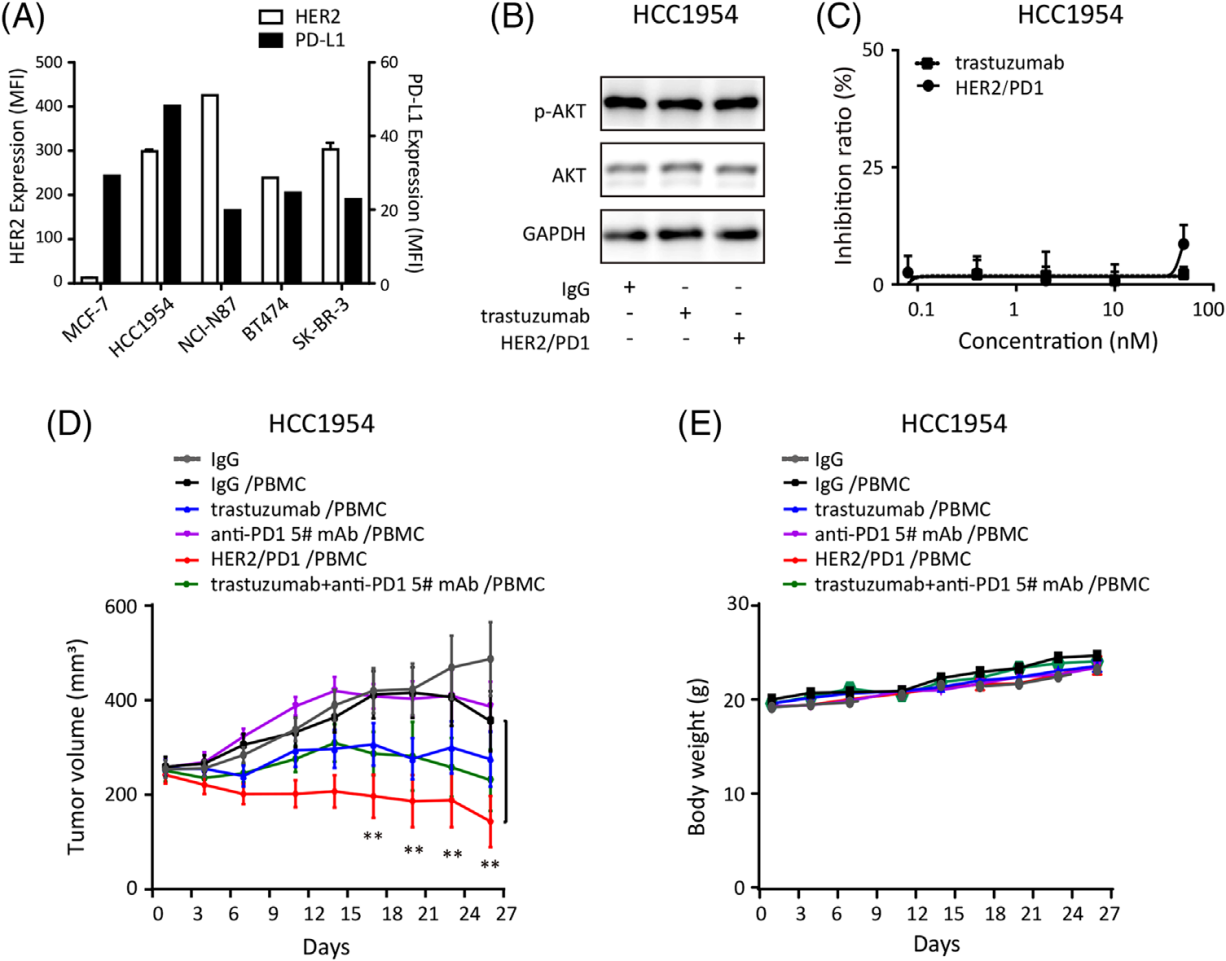
Anti‐tumour effects of HER2/PD1 BsAb in HCC1954 tumour models. (A) Expression levels of HER2 and PD‐L1 on tumour cells were detected by flow cytometry. (B) Neither trastuzumab (5 nM) nor HER2/PD1 BsAb (5 nM) could inhibit the AKT phosphorylation in HCC1954 cells after 6 h treatment. (C) Neither trastuzumab nor HER2/PD1 BsAb could inhibit the proliferation of HCC1954 cells after 72 h treatment. (D) Anti‐tumour activities of antibodies in HCC1954 xenograft tumour model in human PBMC‐infused NCG mice (*n* = 8). Note that 10 mg/kg for BsAb and 7.5 mg/kg for mAbs were in equal molar dose. (E) Body weights of NCG mice. Results were shown as mean ± SEM. ^*^ *p *< .05, ^**^ *p *< .01, ^***^ *p *< .001 by two‐way ANOVA

HER2/PD1 BsAb in IgG1 subclass may launch ADCC and CDC attacks on HER2‐expressing tumour cells as well as PD1‐expressing activated T cells. In contrast, BsAb in IgG4 can spare activated T cells but would be unable to engage tumour‐targeted ADCC and CDC. We next compared HER2/PD1 BsAbs in IgG1 and IgG4 subclasses. BsAb in IgG4 exhibited about half the ADCC effects of that BsAb in IgG1 and trastuzumab did on NCI‐N87 and BT474 cells (Figure 4A,B), while only slight differences were observed on SK‐BR‐3 cells (Figure 4C). In contrast, BsAb in IgG1, but not in IgG4 subclass, exhibited potent cytotoxicity on activated CD4^+^ T cells (Figure 4D), whose PD1 expression levels were confirmed by flow cytometry (Figure 4E). Interestingly, neither BsAbs triggered significant ADCC effects on activated CD8^+^ T cells that expressed PD1 (Figure S5A,B). The difference between the two main T cell subsets in response to ADCC merits additional investigation, and it is speculated that activated CD8^+^ T cells have inhibitory mechanisms to avoid ADCC. The advantages of BsAb in IgG4 over IgG1 subclass were further investigated in a HER2‐positive NCI‐N87 gastric tumour model inoculated into NCG mice injected with human PBMCs. Interestingly, BsAb in IgG4 showed more potent anti‐tumour effects than that in IgG1 subclass (Figure 4F), without impacting the body weight (Figure 4G). Therefore, the importance of protecting T cells may outweigh that of tumour‐targeted ADCC and CDC in cancer treatment by BsAb. Taken together, HER2/PD1 BsAbs in IgG4 may provide a mechanism of action different from BsAbs in IgG1 and may offer patients additional clinical benefits.

In summary, HER2/PD1 BsAb in IgG4 subclass was designed and optimized, which retained the activities of both parental mAbs and could link HER2‐ and PD1‐expressing cells. In a trastuzumab‐resistant HCC1954 tumour model, BsAb was superior to respective mAb and combination therapies. More importantly, it avoided ADCC toxicity to activated CD4^+^ T cells and was more effective than BsAb in IgG1 subclass *in animal model*. Collectively, HER2/PD1 BsAb in IgG4‐scFv format may potentially be a more effective treatment for overcoming drug resistance to HER2‐targeted therapies in clinic.

**FIGURE 4:**
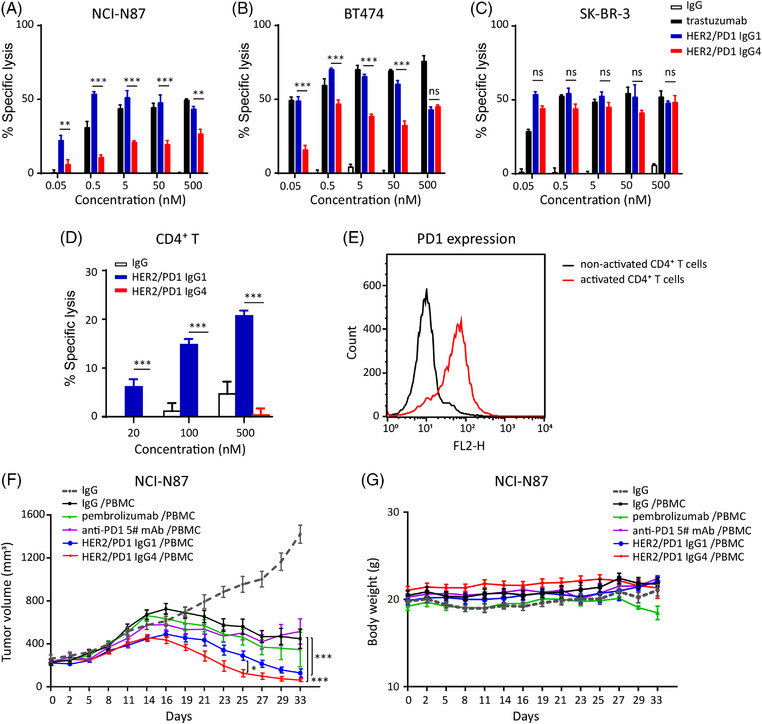
Comparison of anti‐tumour activities between HER2/PD1 BsAb in IgG1 and IgG4 subclass. The ADCC effects of BsAbs in different co‐culture systems at PBMC effector cells to target cells (A) NCI‐N87, (B) BT474, (C) SK‐BR‐3 or (D) activated CD4^+^ T cells with ratio of 50:1. (E) Purified CD4^+^ T cells were isolated and activated for 72 h, then PD1 expression of CD4^+^ T cells were detected by flow cytometry. Naïve CD4^+^ T cells were used as control. (F) Anti‐tumour activities of HER2/PD1 BsAbs (10 mg/kg) in IgG1 and IgG4 subclass on NCI‐N87 xenograft tumour model in human PBMC‐infused NCG mice (*n* = 8). (G) Body weights of the nude mice. Results were shown as mean ± SEM. ^*^ *p *< .05, ^**^ *p *< .01, ^***^ *p *< .001 by two‐way ANOVA

## CONFLICT OF INTEREST

Chunhe Wang received stipends from Dartsbio Pharmaceuticals, Ltd. and Yi‐Li Chen from Shanghai Mabstone Biotechnology, Ltd., respectively. The rest of authors declare no conflict of interest.

## Supporting information

Supporting Information
