# Supplementary material for: HER2/PD1 bispecific antibody in IgG4 subclass with superior anti‐tumour activities
Source: Clin Transl Med. 2022 Apr 5;12(4):e791. doi: 10.1002/ctm2.791 (PMC8982313; doi:10.1002/ctm2.791)
Supplement: Supplementary file 1 — Supporting Information [file CTM2-12-e791-s001.pdf]

Supplementary Materials for

HER2/PD1 bispecific antibody in IgG4 subclass with superior anti-tumour activities

This file includes:

Figure S1 to S5

Table S1

Materials and Methods

References

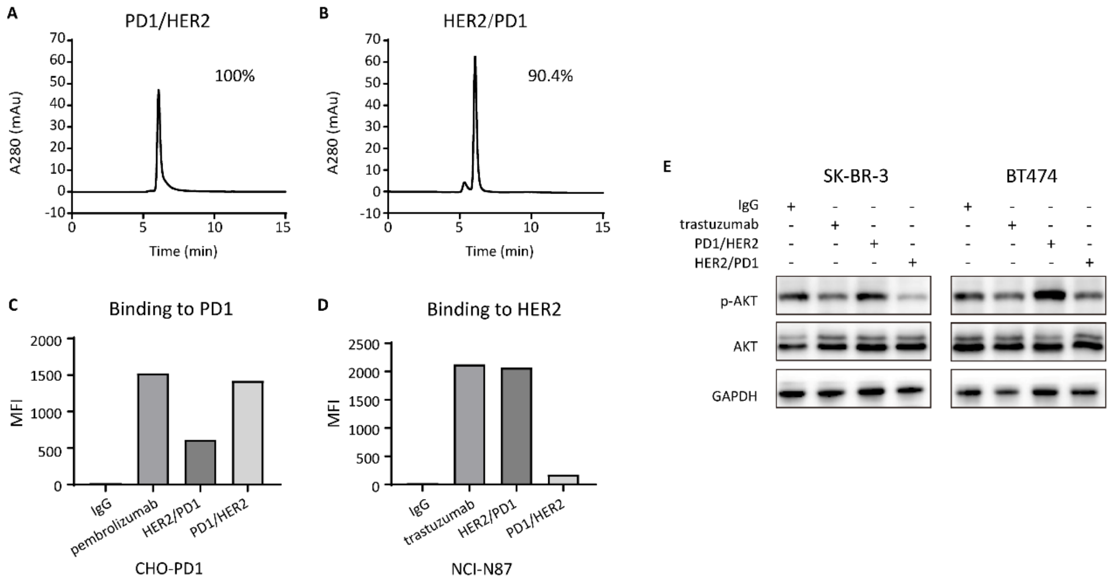

Figure S1. The design, expression and selection of IgG-scFv BsAbs. Purity of (A) PD1/HER2 and (B) HER2/PD1 BsAbs. (C) BsAbs (50 nM) bound to PD1 on the surface of CHO-PD1 cells as analysed by flow cytometry. (D) BsAbs (50 nM) bound to HER2 on the surface of SK-BR-3 cells as analysed by flow cytometry. (E) Inhibition of HER2/PD1 BsAb (50 nM), PD1/HER2 BsAb (50 nM) and trastuzumab (50 nM) on AKT phosphorylation in HER2-positive breast tumour cells after 6 h treatment. The experiments were repeated 3 times. Results were shown as mean ± SEM. $^{*}p < 0.05$, $^{***}p < 0.001$ by one-way ANOVA.

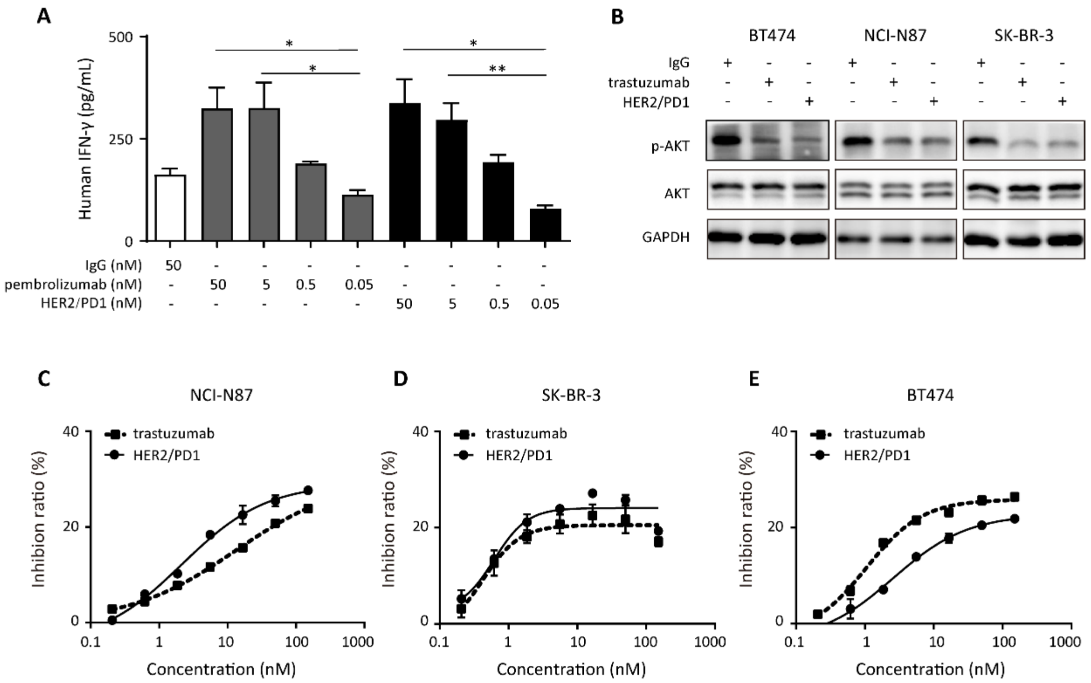

Figure S2. HER2/PD1 BsAb activated T cells and inhibited the proliferation of HER2-positive cancer cells. (A) BsAb increased IFN-γ production in a dose-dependent manner and showed comparable efficacy to pembrolizumab. Dendritic cells (DCs) were induced from $CD14^+$ cells *in vitro* for 5 days and matured for another 2 days. Then, mature DCs (1×10^4) and allogenic $CD4^+$ T cells (1×10^5) were co-cultured for 5 days with or without indicated concentrations of antibodies, and IFN-γ in culture supernatants was detected by ELISA. (B) Inhibition of AKT phosphorylation by antibodies in HER2-positive cancer cells. Cells treated with 5 nM of antibodies were assayed for p-AKT by western blot. (C-E) BsAb inhibited the proliferation of these HER2-positive cancer cells with a similar potency to trastuzumab. The experiment was carried out in triplicate. NCI-N87, SK-BR-3 and BT474 cells were treated with various concentrations of trastuzumab or HER2/PD1 BsAb for 72 h, and the cell proliferation was determined by CCK-8 assay. Data were shown as mean ± SEM. $^{*}p < 0.05$, $^{**}p < 0.01$, by one-way ANOVA.

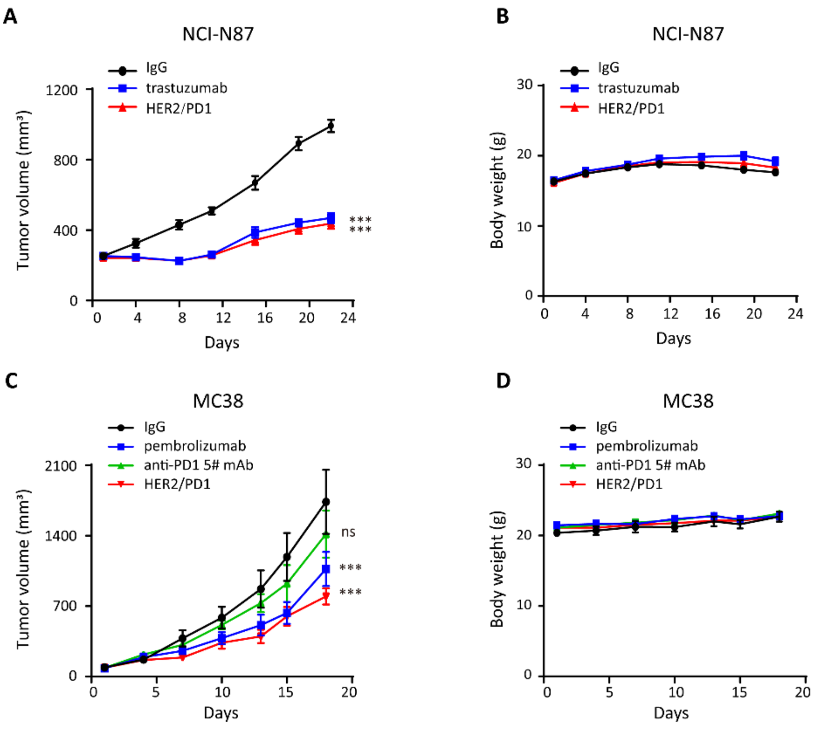

Figure S3. Antitumour effects of HER2/PD1 BsAb in various tumour models. (A) HER2-positive NCI-N87 xenograft tumour model in nude mice (*n* = 10) was treated with equal molar dose of BsAb (10 mg/kg), trastuzumab (7.5 mg/kg) or human IgG (7.5 mg/kg), twice a week. (B) Body weights of the nude mice. (C) MC38 syngenetic tumour model in humanized PD1 C57BL/6 mice (*n* = 5) was treated with equal molar dose of BsAb (10 mg/kg), pembrolizumab (7.5 mg/kg) or human IgG (7.5 mg/kg), twice a week. (D) Body weights of C57BL/6-hPD1 mice. Results were shown as mean ± SEM. $^{***}p < 0.001$ by two-way ANOVA.

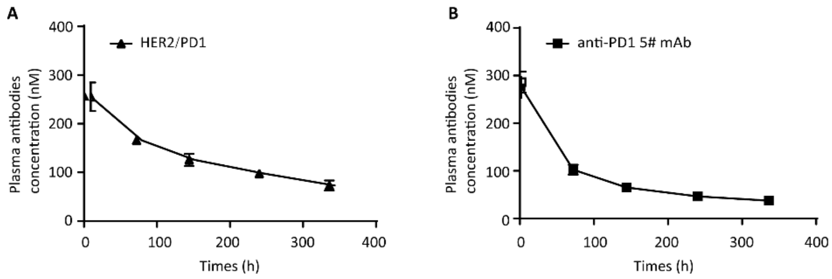

	HER2/PD1	anti-PD1 5# mAb
$T_{1/2}$ (h)	244	240
MRT (h)	334	282
$AUC_{0\text{-}t}$ (h*nM)	44700	29200
CL (μg/(h*nM)/kg)	0.142	0.178
Vss (mg/nM/kg)	0.0475	0.05

Figure S4. Pharmacokinetics of HER2/PD1 BsAb and anti PD1 5# mAb in BALB/c nude mice. Plasma samples were collected from BALB/c-nu nude mice (*n* = 3 per dose group). $T_{1/2}$, half-life; MRT, mean retention time; $AUC_{0\text{-}t}$, area under the curve from time 0 to the last sampling time; CL, clearance; Vss, volume of distribution at steady state.

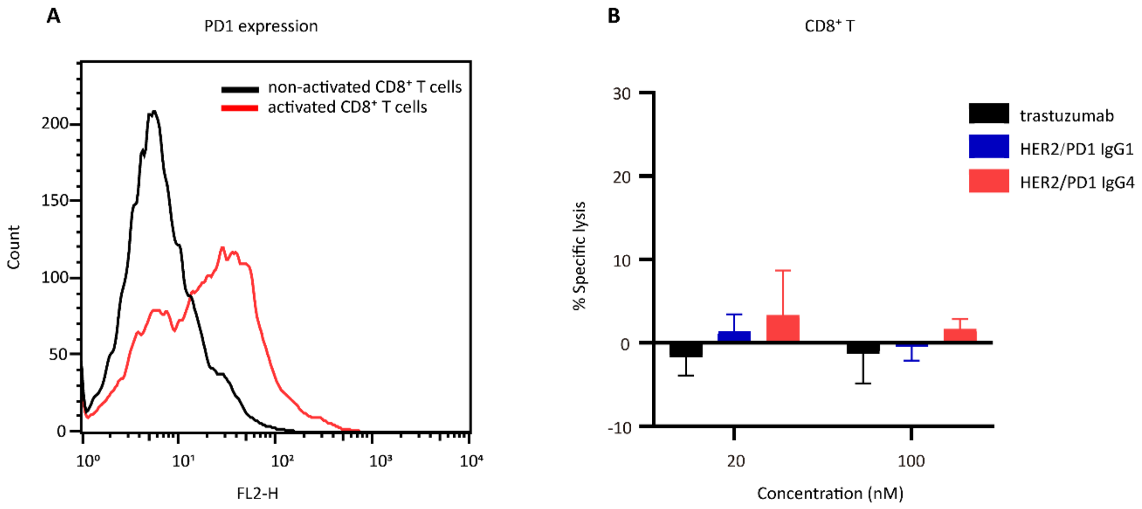

Figure S5. Both trastuzumab and two HER2/PD1 BsAbs showed no significant ADCC effects on activated CD8$^+$ T cells. (A) Purified CD8$^+$ T cells were isolated and activated for 72 h, then PD1 expression of CD8$^+$ T cells were detected by flow cytometry. Naïve CD8$^+$ T cells were used as control. (B) The ADCC effect of BsAbs and trastuzumab in co-culture systems at an effector (PBMCs) to target cells (activated CD8$^+$ T cells) with the ratio of 50:5. Results were shown as mean ± SEM.

Table S1. Purity and antigen-binding affinity of anti-PD1 mAbs

Antibody	Purity (%)	IC_{50} (nM)	KD (nM)	EC_{50} (nM)
HG 1#	98.9	3.89	8.08	0.047
2#	96.3	3.17	1.78	0.021
3#	91.8	2.67	3.25	0.059
4#	93.4	2.56	2.30	0.051
5#	97.0	2.50	1.40	0.046
6#	94.1	2.94	2.25	0.037
7#	89.5	4.06	3.94	0.061
8#	98.4	3.50	5.46	0.071
9#	98.1	2.22	3.53	0.046

IC_{50}: half maximal inhibitory concentration of neutralizing activity;

KD: the equilibrium dissociation constant between the antibody and its antigen;

EC_{50}: half-maximal effective concentration of binding activity.

Materials and Methods

Cell culture

NCI-N87, SK-BR-3, BT474, HCC1954, MCF-7, MC38 and EMT6 cells were purchased from ATCC (Manassas, VA). NCI-N87 and HCC1954 cells were cultured in RPMI-1640 medium (Gibco, CA) supplemented with 10% fetal bovine serum (FBS) (Gibco, CA). SK-BR-3 cells were cultured in DMEM/F-12 medium supplemented with 10% FBS. BT474 cells were cultured in DMEM medium (Gibco, CA) supplemented with 10% FBS and 20 μM of sodium pyruvate (Gibco, CA). MCF-7 and MC38 cells were cultured in DMEM medium supplemented with 10% FBS. EMT6 cells were cultured in Waymouth MB752/1 medium (Gibco, CA) supplemented with 10% FBS. CHO-PD1 and HEK293F cells were purchased from National Collection of Authenticated Cell Cultures (Shanghai, China). CHO-PD1 cells were cultured in CD CHO medium (Gibco, CA) supplemented with 10 mM glutamine (Gibco, CA) and 0.7 mg/ml G418 sulphate (Gibco, CA). HEK293F cells were cultured in FreeStyle™ 293 Expression Medium (Gibco, CA). Cells were cultured at 37 °C in a humidified atmosphere containing 5% CO_2. Human peripheral blood mononuclear cells (PBMCs) were purchased from MiaoTong Biotechnology (Shanghai, China).

Generation of murine anti-PD1 antibody 5#

The murine version of anti-PD1 antibody 5# was generated by mouse immunization and hybridoma technology. Six-week-old female BALB/c mice were immunized with hPD1 overexpressing HEK293T cells by standard vaccination procedures. The positive clones were selected by an indirect ELISA assay coated with recombinant human PD1 protein, and subjected to colonized culture for obtaining stable single clones. Anti-PD1 antibodies were first evaluated by testing their binding reactivity with a recombinant hPD1 protein and then examined their ability to block the interaction between PD1 and PD-L1.

Humanization of murine anti-PD1 antibody 5# by framework shuffling

Murine hybridoma cells secreting monoclonal antibody (mAb) against human PD1 were prepared as described above. Total RNA was isolated from hybridoma cells and variable region genes of PD-L1 antibody were obtained by degenerate polymerase chain reaction (PCR). Anti-PD1 mAb was humanized with framework shuffling strategy with human heavy and light germline genes. The procedure was carried out essentially as described previously[1] and resulted in the discovery of humanized antibody molecule anti-PD1

antibody 5# exhibiting excellent thermal stability, binding affinity and specificity as determined by ELISA.

BsAb engineering and expression

Anti-HER2 antigen-binding fragment (Fab), V_H and V_L domain sequences were adopted from trastuzumab. Anti-PD1 Fab, V_H and V_L domain sequences were from anti-PD1 antibody 5#. The Fc (Fragment, crystallizable) domain sequence of IgG1 was identical to that of trastuzumab. The Fc domain of IgG4 contains S288P mutation[2] to reduce Fab-arm exchange. V_H and V_L domains of scFv were connected by a $(GGGGS)_4$ linker. The scFv was connected to the C-terminus of IgG heavy chain (HC) by a GGGGSGGGGTGGGGS linker. Plasmids encoding BsAbs were transiently co-transfected in HEK293 cells and the cell culture supernatants were harvested and purified by AT Protein A Diamond (BestChrom, Shanghai, China) affinity chromatography.

Thermal stability assay

Differential Scanning Fluorimetry (DSF) was used to measure the thermal stability of antibodies. 1 μl of SYPRO® Orange (Thermo Fisher Scientific, Waltham, MA) 250 × working solution was added to 24 μl of PBS-diluted antibody at 20 μM. Relative fluorescence units (RFU), dRFU/dT curve and T_m were obtained on a CF × 96 Touch qPCR machine (Bio-Rad, Hercules, CA). Thermal stabilities of antibodies were also measured by stress studies. After antibodies (10 mg/ml) were heated at 60 °C for 1 h, the percentage of monomer was measured by analytical size exclusion chromatography (SEC)-HPLC. ANS (8-anilino-1-naphthalenesulfonic acid, #10417, Sigma) was used to monitor protein conformational changes by binding to the hydrophobic regions of a protein. After being heated at 60 °C for 1 h, samples were mixed with an equal volume of diluted ANS at room temperature, and the fluorescence emission spectrum was collected from 390 to 650 nm with a step size of 3 nm following excitation at 360 nm.

Flow cytometry

5×10^5 cells were incubated with 50 nM antibodies in 500 μl volume at 37 °C for 1 h. Cells were washed twice with ice-cold phosphate-buffered saline (PBS) and then incubated with 1 μg of PE-anti-human IgG Fc antibody in 500 μl volume for 15 min in the dark. Cells were analysed on a flow cytometer (FACScan™, Becton Dickinson, San Diego, CA).

Western blot

NCI-N87, BT474, SK-BR-3 or HCC1954 cells were lysed with lysis buffer (1× loading buffer containing 2% SDS (Sodium Dodecyl Sulfate), 0.01% bromophenol blue, 50 mM Tris-HCl, 10% glycerol and 100 mM DTT). Cell lysates were detected by anti-AKT (Cell Signaling Technology) or anti-p-AKT (Ser473, Cell Signaling Technology) primary antibodies and then goat anti-rabbit IgG or goat anti-mouse IgG (Jackson ImmunoResearch) secondary antibodies conjugated with HRP using a standard western blot protocol.

Biolayer interferometry (BLI)

Binding affinities were measured by BLI using Octet Red96® (Fortebio, Menlo Park, PA). Antibodies (10 μg/ml) were captured by protein A biosensors. The baseline was established by PBS for at least 60 s, followed by capture of antibodies until the BLI signal increased up to approximately 1 nm. Subsequently, a second baseline was acquired in the buffer for 60 s, followed by incubation with 50 nM of PD1 (C-6×His) (Novoprotein) or HER2 (C-6×His) (Novoprotein) for 180 s. The data were analysed by the software provided by the manufacturer.

BsAb binding ELISA

96-well Immuno-plates (Greiner, Monroe, NC) were coated with 1 µg/ml HER2 (C-6×His) proteins (Novoprotein) for 1.5 h at 37 °C. After the plates were blocked at room temperature for 1 h with 1% casein (Thermo Fisher Scientific), serial dilutions of antibodies were added and incubated for 1 h at 37°C before the plates were washed twice with PBS containing 0.05% Tween-20. 1 µg/ml of biotinylated PD1 protein were added to the plates and incubated for 1 h. After the plates were washed twice, streptavidin-HRP (Thermo Fisher Scientific) was added and incubated for 1 h. After the plates were washed twice, TMB substrate (3,3',5,5'-Tetramethylbenzidine, Thermo Fisher Scientific) and 2 M of H_2SO_4 were added and OD_{450} values were measured by a SpectraMax M5e microplate reader (Molecular Devices, San Jose, CA).

PD1/PD-L1 neutralization assay

The HTRF (Homogeneous Time-Resolved Fluorescence) PD1/PD-L1 Binding Assay (Perkin Elmer) was performed to measure the interaction between PD1 and PD-L1 proteins. According to the manual, the interaction between Tag1-PD-L1 and Tag2-PD1 was detected by anti-Tag1-Europium (HTRF donor) and anti-Tag2-XL665 (HTRF acceptor). When the donor and acceptor antibodies were brought into proximity due to

PD-L1 and PD1 binding, excitation of the donor antibody triggered fluorescent resonance energy transfer (FRET) towards the acceptor antibody, which in turn emitted specifically at 665 nm. This specific signal was directly proportional to the extent of PD1/PD-L1 interaction. Thus, antibodies blocking PD1/PD-L1 interaction would cause a reduction in HTRF signal. At first, 2 µl of antibodies, 4 µl of Tag1-PD-L1 protein and 4 µl of Tag2-PD1 protein were added into 96-well plates. After 15 min incubation at room temperature, 10 µl of pre-mixed anti-Tag1- Europium and anti-Tag2-XL665 were added to the plates. After 2 h, samples were read on the HTRF compatible reader (Spark Cyto, TECAN), and the ratio of the acceptor and donor emission signals were calculated as: Ratio = $(Signal_{665\ nm} / Signal_{620\ nm}) \times 10^4$.

ADCC assay

ADCC was measured with a DELFIA Cell Cytotoxicity Kit (Perkin Elmer, Waltham, MA). PBMCs were incubated overnight with 100 ng/ml of IL-2 (Novoprotein) and used as effector cells. Highly purified $CD4^+$ T and $CD8^+$ T cells from PBMCs were isolated with EasySep™ Human $CD4^+$ T or $CD8^+$ T Cell Isolation Kit (Stemcell Technologies, Vancouver, Canada) and activated for 72 h using one loaded Anti-Biotin MACSiBead Particle per two T cells (Miltenyi Biotec, Bergisch Gladbach, Germany) and 50 ng/ml of IL-2. HER2-expressing tumour cells, activated $CD4^+$ T and $CD8^+$ T cells labelled with BATDA reagent were used as target cells. The effector-to-target cell ratio was 50:1 or 50:5. Cells were incubated for 2-4 h at 37°C. 20 µl of the supernatant was transferred to a flat-bottom plate before 200 µl of Europium Solution were added. After incubation for 15 min at room temperature on the DELFIA Plateshake, the fluorescence was measured on a time-resolved fluorometer (Spark Cyto, TECAN, Männedorf, Switzerland).

Cell proliferation assay

NCI-N87, BT474, SK-BR-3 and HCC1954 cells were seeded at 3,000 cells/100 µl/well in 96-well culture plates, and incubated overnight at 37°C with 5% CO_2. After 24 h, cells were incubated with serially diluted HER2/PD1 BsAb or trastuzumab. The plates were incubated at 37°C for 72 h, and 10 µl of CCK8 (Cell Counting Kit-8, Vazyme, Nanjing, China) were added. OD_{450} values were read on a SpectraMax plus 384 (Molecular Devices) within 2 h.

Mixed lymphocyte reaction (MLR) assays

$CD4^+$ T cells and $CD14^+$ cells were isolated from frozen PBMCs from different donors by EasySep™ Human $CD4^+$ T Cell Isolation Kit (Stemcell Technologies) and CD14 MicroBeads human lyophilized kit (Miltenyi Biotec). DCs were generated by culturing $CD14^+$ cells *in vitro* for 5 days with 500 ng/ml of IL-4 and 500 ng/ml of GM-CSF (Granulocyte-Macrophage Colony Stimulating Factor). Mature DCs were induced by TNF-α (30 ng/ml), IL-1β (300 ng/ml), IL-6 (300 ng/ml) and PGE2 (3 μg/ml) for 2 days. All stimulating factors were purchased from Novoprotein. $CD4^+$ T cells (1×10^5) and allogeneic DCs (1×10^4) were co-cultured with antibodies. After 5 days, IFN-γ in culture supernatants was detected by ELISA.

Cell coupling assay

HER2-expressing NCI-N87 tumour cells were labelled with carboxyfluorescein succinimidyl ester (CFSE) (Thermo Fisher Scientific) and CHO-PD1 cells were labelled with cell proliferation dye eFluor 670 (Thermo Fisher Scientific). After washing, cells were suspended and mixed with an equal volume of 25 nM of antibodies or a combination of 25 nM of anti-HER2 and 25 nM of anti-PD1 to a total volume of 200 μl, followed by incubation on ice for 30 min. After washing, the resuspended cell mixtures were assayed by flow cytometry (CytoFLEX, Beckman Coulter, Brea, CA) for the percentage of double-positive cell population.

Xenograft tumour models

All animal experiments were performed according to the institutional ethical guidelines on animal care and approved by the Institute Animal Care and Use Committee at Shanghai Institute of Materia Medica. Mice used in the experiments were 6 to 8 weeks old females and were randomly divided into different groups. BALB/c-nu nude mice were purchased from Vital River Laboratory Animal Technology (Beijing, China). BALB/c-hPD1 (human PD1 "knock-in") mice were from Biocytogen (Beijing, China). C57BL/6-hPD1 (human PD1 "knock-in") mice and NCG mice were from GemPharmatech (Nanjing, China).

NCI-N87 cells (5×10^6) were injected subcutaneously in the armpit of BALB/c-nu nude mice (3 groups, n = 10 per group). MC38 (5×10^5) were injected subcutaneously into the armpits of C57-hPD1 mice (4 groups, n = 5 per group). HCC1954 cells (1×10^7) or NCI-N87 cells (5×10^6) were injected subcutaneously in the armpits of NCG mice (6 groups, n = 8 per group) before PBMCs (1×10^6 or 5×10^5) were *in situ* injected. Mice were treated

with intraperitoneal injections of antibodies every 3 days for nine times with an equal molar dose of 10 mg/kg (BsAb) or 7.5 mg/kg (mAbs). Tumour diameters were recorded with an electronic calliper every 3 days and tumour volume was calculated with the following formula: tumour volume (mm^3) =0.5 × length (mm) × [width (mm)]^2. Body weights were measured by an electronic scale.

Pharmacokinetic study in mice

In a single-dose pharmacokinetic study, BALB/c-nu nude mice were intravenously injected with 10 mg/kg of HER2/PD1 BsAb in IgG4 subclass. Plasma samples were collected in K3EDTA tubes at 2, 72, 144, 240 and 336 h after the administration from the orbit (n = 3 per dose group, per time point) by centrifuging at 3,000×g for 5 min at 4 °C, and supernatants were stored at -80 °C until analysis. The OD_{450} values of a set of HER2/PD1 BsAb concentration standards were established by ELISA assay with PD1-Fc protein (Sino Biological, Beijing, China) coating on the plate and detected by biotinylated anti-IgG (#ab97223, Abcam) using SpectraMax plus 384 (Molecular Devices), which was used to plot a standard curve. The plasma concentrations of antibodies were calculated from the standard curve using four-parameter curve fit. The pharmacokinetic parameters were calculated by Certara Phoenix WinNonlin 8.1.

Statistical analysis

All data were presented as mean ± standard error of mean (SEM). P values were calculated using one-way ANOVA or two-way ANOVA multiple comparison test. p < 0.05 was considered as statistically significant ($^{*}p < 0.05$, $^{**}p < 0.01$, $^{***}p < 0.001$, ns: not significant).

References

1. Wec Anna Z, Nyakatura Elisabeth K, Herbert Andrew S, et al. A "Trojan horse" bispecific-antibody strategy for broad protection against ebolaviruses. *Science* 2016; 354(6310): 350-4.
2. Labrijn AF, Buijsse AO, van den Bremer ETJ, et al. Therapeutic IgG4 antibodies engage in Fab-arm exchange with endogenous human IgG4 in vivo. *Nature Biotechnology* 2009; 27(8): 767-U122.
